# Motion Magnification of Vibration Image in Estimation of Technical Object Condition-Review

**DOI:** 10.3390/s21196572

**Published:** 2021-09-30

**Authors:** Michał Śmieja, Jarosław Mamala, Krzysztof Prażnowski, Tomasz Ciepliński, Łukasz Szumilas

**Affiliations:** 1 Faculty of Technical Sciences, University of Warmia and Mazury in Olsztyn, 46 A, Słoneczna St., 10-710 Olsztyn, Poland; 2 Department of Mechanics and Structural Engineering, Faculty of Civil Engineering and Architecture, Opole University of Technology, 45-061 Opole, Poland; j.mamala@po.edu.pl (J.M.); k.praznowski@po.edu.pl (K.P.); 3 I-Care Polska Sp. z o.o., ul. Puszkarska 9, 30-644 Kraków, Poland; tomasz.cieplinski@icareweb.com (T.C.); lukasz.szumilas@icareweb.com (Ł.S.)

**Keywords:** motion magnification, mechanical vibration, visual vibration estimation, machine condition monitoring

## Abstract

One of the most important features of the proper operation of technical objects is monitoring the vibrations of their mechanical components. The currently significant proportion of the research methods in this regard includes a group of research methods based on the conversion of vibrations using sensors providing data from individual locations. In parallel with the continuous improvement of these tools, new methods for acquiring information on the condition of the object have emerged due to the rapid development of visual systems. Their actual effectiveness determined the switch from research laboratories to actual industrial installations. In many cases, the application of the visualization methods can supplement the conventional methods applied and, under particular conditions, can effectively replace them. The decisive factor is their non-contact nature and the possibility for simultaneous observation of multiple points of the selected area. Visual motion magnification (MM) is an image processing method that involves the conscious and deliberate deformation of input images to the form that enables the visual observation of vibration processes which are not visible in their natural form. The first part of the article refers to the basic terms in the field of expressing motion in an image (based on the Lagrangian and Eulerian approaches), the formulation of the term of optical flow (OF), and the interpretation of an image in time and space. The following part of the article reviews the main processing algorithms in the aspect of computational complexity and visual quality and their modification for applications under specific conditions. The comparison of the MM methods presented in the paper and recommendations for their applications across a wide variety of fields were supported with examples originating from recent publications. The effectiveness of visual methods based on motion magnification in machine diagnosis and the identification of malfunctions are illustrated with selected examples of the implementation derived from authors’ workshop practice under industrial conditions.

## 1. Introduction

Monitoring vibration processes of technical objects is an important part of their use. This applies to both the periodical assessment of the technical condition of a machine or structure, subject to natural wear. The particular cases in which the relationship between an object’s operating parameters and the events, and conditions which have occurred is not well documented. In such an approach, a vibration signal acts as a diagnostic symptom which informs about adverse or dangerous changes within the object’s structure or interactions with the environment. The observation of vibrations is not, however, limited only to regarding them as the so-called residual process. In many machines, such as conveyors or vibrating tables, vibrations serve as the main working motion. In such cases, the current vibration picture enables, for example, the optimal adjustment of a device to the load or performance nature.

The classical tools currently used for vibroacoustic testing are accelerometric transducers (piezoelectric, MEMS), whose output signal transmits information about absolute accelerations at locations corresponding to their mounting point [1]. Where a non-contact measurement is required, eddy-current sensors [2] based on inductive or laser interactions relying on triangulation methods for distance measurement, or velocity determination from the Doppler effect, are increasingly being used [3]. The values obtained from transducers are used directly as required or subjected to further transformations to obtain selected kinematic or geometric parameters. These values, in combination with modern hardware and software computing tools, currently enable a very flexible description of vibration effects. Traditional diagnostic inference methods based on the data provided as a result of acceleration, velocity and displacement measurements using the aforementioned sensors are mainly local in scope and are normally limited to individual points. This is an important constraint as it regards applications on objects characterized by their high complexity, in terms of the number of interacting components with multiple degrees of freedom, or the complexity of strains. Solutions applied in such situations, which involve the multiplication of measuring points, are associated with the extension of the measurement chain and a significant increase in the research performance costs. In many cases, theoretical knowledge of the objects’ structure and the parameters describing its dynamic properties enable the reconstruction of the cause-and-effect chain between the readings of an individual sensor, and the occurring interactions by simulation. However, the final effect of such an approach can significantly differ from the image obtained through direct measurements. Due to the commonness of diagnostic methods based on the analysis of vibrations generated by an object, they are often performed routinely based on the adopted standard procedures. In many cases, however, the selection of the measuring range or sensor location requires additional assembly operations or multiple recordings, which prolongs the time of identifying the object’s condition. An example of the problem and solutions related to high spatial resolution structural vibration in the presence of large rigid-body motion are presented in [4,5]. There are also other constraints and disadvantages regarding the use of classical measuring sensors, e.g., adverse environmental conditions (high temperatures, humidity), difficulties in installing the sensor on an object, or the effect of the sensor’s weight on the dynamic parameters of the objects being tested [6]. Exemplary comparison of constrains of classical sensor (accelerometer) and visual based sensor (camera) is presented in Table 1.

Modern image analysis and processing algorithms extend the existing possibilities of vibration observation. An approach based on the triangulation method and point-tracking for the observation of static and dynamic strains of a civil structure is presented in [7]. A significant group of visual techniques that meets such important advantages as the possibility for simultaneous recording of data on the location, velocity and acceleration of multiple points, as well as the non-contact mode of operation to the so-called motion magnification (MM). An example of the comparison of the effects of multipoint observation using MM and classic sensors in the application for verifying the arm of a cobot model is presented in [8].

The following part of the article provides the basic motion magnification (MM) assumptions and methods on which the research using the so-called motion magnification is currently based, and selected examples of the application of these tools for the diagnostics of equipment operating under industrial conditions. Vibration measurement, using sensors and tools processing the read off signal to a form interpretable to a researcher, is an indirect method for observing physical processes taking place. This approach allows one to exceed the human’s natural threshold of visual perception, above which minimal changes within the observer’s field of vision can be reflected in consciousness, or “consciously seen”. It should be noted that the expression “minimal” does not apply to the actual geometric quantities that locate an object in its environment. The final results of this approach are expressed in a quantitative manner, and the visualizations created on this basis take the form of graphs (histograms, charts, phasors, etc...). An alternative, direct way of representing the information of an object’s vibrations as its displacements or mutual displacements of its selected points (deformations) over time may be descriptive, based directly on the perceived actual image only to a limited extent. Certain considerations about the spatio-temporal data visualization are provided in [9]. Assuming that the identification of vibrations is not an end in itself, and the aim is to gain information on potential results or causes of this phenomenon, a direct approach can prove much more effective. An example of this can be a descriptive assessment of the wind, based on the observed current state of the sea surface carried from a ship’s deck [10].

For many technical objects, a readable visualization of vibrations allows much quicker and more intuitive conclusions to be drawn rather than complex measurement procedures. Experiments in the field of image analysis and processing, as well as a significant increase in processor computational resources in commonly available electronic devices, allowed operations to be undertaken to provide an answer to the question as to how to expand the range of possibilities for visual observation of vibrations, while taking account of the additional element between the observer and the moving object in the form of a camera. A raw image sequence recorded on the camera sensor contains information about the positions of a 3D object’s points projected onto 2D planes in subsequent moments of time. Small differences between adjacent images, corresponding to the object’s movement, are not noticed by the observer when reproducing the original recording. For obvious reasons, modification of the natural way of seeing is not possible. Therefore, to achieve the desired effect, techniques have been developed for transforming a set of images recorded by a camera so that significant differences between the changing frames of the images that reach the observer are located above their visual perception threshold. A shift of image changes included in video sequences, corresponding to the movements of the object under observation, into the area of visual perception is carried out through the “enhancement of motion” operations, known in the literature as motion magnification or motion microscopy [11].

## 2. Optical Flow

To reflect the three-dimensional movement of an object in relation to the observer, it is projected onto a two-dimensional plane of a camera sensor in subsequent moments of time. Consequently, a set of images within a three-dimensional space spread over a single time coordinate and two spatial coordinates is obtained. This set, in a form of a stack of consecutive images, is referred to as a space-time image, and may be represented as an image cube [12].

Individual image components in the slices of this cube are characterized by brightness, which indicates image irradiance [13]. The brightness pattern is the distribution of irradiance in the image or, in other words, the distribution of apparent velocities of movement of brightness patterns in an image. The differences between adjacent images approximate discrete image displacement in time as instantaneous image velocities called optical flow field or image velocity field [14]. Motion appears as orientation in space–time images. The term optical flow is an analogy to the methods for describing the mediums used in fluid mechanics in the Lagrangian or Eulerian approaches. Even though, from the perspective of information on motion dynamics, both approaches are equivalent, the principles of their implementation differ significantly.

**Figure 1:**
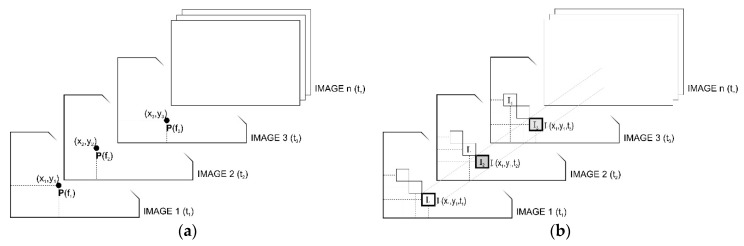
Lagrangian (**a**) and Eulerian (**b**) approach to description of the optical flow.

In the Lagrangian approach, the proper subject of observation is the points or selected areas of the image, as shown in Figure 1. Along the time axis, consecutive coordinates of point P: (x_1_,y_1_), (x_2_,y_2_), (x_3_,y_3_), … (x_n_, y_n_) are tracked in consecutive frames of Image_1, Image_2, …, Image_n.

Contrary to the Lagrangian method, the Eulerian method is focused on the pixel intensity variable over time in the fixed location. I(x_1_,y_1_,t_1_) = I_1_, I(x_1_,y_1_,t_2_) = I_2_,…, I(x_1_,y_1_,t_n_) = I_n_. The selection of the Lagrangian method implies the need to extract a specific object from an image, to be subsequently subjected to tracking. As for the Eulerian method, the “image content” is, from the processing perspective, insignificant. As far as the motion observation issue is concerned, the Lagrangian approach appears to be much more intuitive because it is consistent with our natural way of seeing where we focus on specific components of the scene being perceived. However, the Eulerian methods are gaining increasing popularity.

Similarly, the concepts of “continuity equation“ were taken from fluid mechanics to define base local constraints on image motion called the “optical flow constraint equation” or the “brightness change constraint equation (BCCE)”, expressed as formula (2). Assuming:

(1) I(x,t)≈I(x+δx, t+δt)

where *I*(*x*,*t*) is the image intensity function, and *δx*, *δt* are displacements in space-time domain. Expanding this in the First Order Taylor series results in Equation (2):

(2) $$\nabla I\cdot V+I_{t}=0$$

where $\nabla I=\left(I_{x},I_{y}\right)$ is the spatial intensity gradient, *V* = (*u*,*ν*) is image velocity, I_t_ is partial derivative of *I*(*x*,*t*).

Equation (2) provides the basis for the calculation of optical flow using Global and Local differential methods. An example of applying BCCE to compute Global optical flow over a large image region with additional smoothness constraint was presented by [13]. The BCCE solution in a local spatial neighborhood with weighted least-squares fit of measurement was implemented by Lucas and Kanade [15]. Another class of optical flow techniques which are of significance in the applications of motion magnification include frequency-based methods [16]. In these methods, the information required for the description of motion is associated with the phase in a local-frequency representation of the image sequence. For this reason, this method is also referred to as the phase-based method [14]. Optical flow constraint equation in a frequency space takes the form of formula 3 [14]

(3) $$v^{T}k+\omega =0$$

where *k*, *ω* is the spatiotemporal frequency (*k*—spatio, *ω*—temporal).

Decomposition of input signal is performed for complex valued output with bandpass velocity-tuned filters. The approach presented by [17] considers the issues of scale, speed and orientation in the image being decomposed.

The out-of-plane vision method proposed in [18], as opposed to the previous ones, refers not to the motion “across the frame” but to the motion in the direction perpendicular to the focusing screen plane (the matrix of camera). The changes in the image result from the object’s motion due to the phenomenon of perspective. The distances between points in the image change as the distance from the lens changes. A simplified description of the phenomenon model was based on the camera obscura (pinhole imaging model). For a description of the optical flow or the experiment results, the methods of the space feature point and the pixel feature point were proposed and compared with the theoretical model results. The visual-based tracking and optical flow methods are widely explored in many fields. Examples of such various approaches is presented in [19] for the assessment of the health status of a tower, based on vibration observations. In [20], the robustness of the optical flow-based methods under the conditions of illumination changes and fog interference is described. The issue of temporal filtering in optical flow estimation in mobile robot navigation is presented in [21]. An alternative to the classic methods for determining the optical flow are methods using artificial intelligence (AI) techniques, e.g., those presented in [22], showing optical flow estimation with deep neural networks. The authors of [23] indicate the reduced user involvement in optical flow processing in this approach, illustrated by an example of civil structure monitoring. An overview of different multipoint synchronous measurements of dynamic structural displacement in civil infrastructure inspection is provided in [24].

## 3. Motion Magnification

The area of motion magnification applications is very wide, and includes fields as distant from each other as, for example, medical diagnostics (mainly due to its non-invasive nature) [25], as well as observations of various technical objects [26]. If we have a formalized recording of information in the form of optical flow on the motion in an image, we can consciously deform it in order to bring out the displacements in such a manner so as to enable the inference about the processes occurring at the objects which are invisible to the naked eye on the direct observation of the video recording.

### 3.1. Lagrangean

An example of the application of the Lagrangian approach for the visualization of deformations invisible in the original recording is provided in [27]. The presented technique groups point with affine trajectories. To this end, characteristic image points are tracked in the time sequence. Their trajectories are clustered into sets of correlated motions. Following interpolation, a dense motion field for each motion cluster, each pixel is assigned to an appropriate motion layer [28]. In this way, a group of pixels is segmented. Motion magnification is achieved by indicating the appropriate layer, multiplying all displacements by the constant factor, and rendering the pixels of each motion layer from back to front. As a result of the translation of the segmented groups of points, holes are formed at the boundary of this area. In the case under consideration, they are filled in using the texture synthesis method [29,30]. From the Lagrangian approach perspective, the characteristic features of the presented method are the extraction of a specific object (a group of points) from the image, and basing further processing on its parameters. A major disadvantage of the presented method is the significant computational complexity which excludes it from applications requiring higher time regimes.

### 3.2. Eulerian Linear

**Figure 2:**
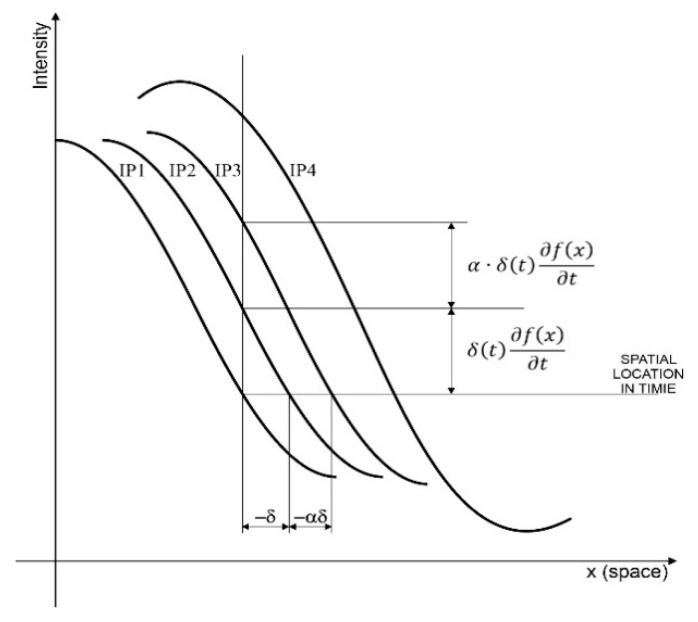
Linear video magnification LVM.

The alternative group of techniques based on the Eulerian description of optical flow currently mainly uses the differential and frequency methods. A brief overview of Eulerian methods is provided in [31]. Presented in [32], the amplification of small motions relies on the first order Taylor expansion. Linear video magnification (LVM) [32] uses the variation of the intensity in pixels on a specific spatial coordinate due to the shift of the object image in the camera’s field of view. In Figure 2, based on the example of the case 1D, the relationships are presented between the shift of the intensity profile in the space and time, based on the assumption of linearity.

The input signal intensity at subsequent moments t and t+1 corresponding to the successive frames was marked in the Figure 2 as the lines IP1 and IP2. With the assumption of linearity, the shift of the highlighted object along the *x*-axis (horizontally) is proportional to its shift (*translation*) along the intensity axis (vertically). Its perceptual effect is the shift of the object image. The amplification in the LVM method being discussed is yielded by the multiplication of the brightness variation in time by the α factor, which results in the line IP3 being obtained. This relationship can be expressed as the following conversions:

(4) I(x,0)=f(x)

Formula (4) express intensity at position x in time = 0

(5) I(x,t)=f(x+δt)

Formula (5) express intensity at position *x* in time = *t*

On the base of the first-order Taylor series expansion about *x*

(6) $$I\left(x,t\right)\approx \;f\left(x\right)+\delta \left(t\right)\frac{\partial f\left(x\right)}{\partial x}$$

(7) $$\hat{I}\left(x,t\right)=I\left(x,t\right)+\alpha \delta \left(t\right)\frac{\partial f\left(x\right)}{\partial x}$$

(8) $$\hat{I}\left(x,t\right)=f\left(x\right)+\delta \left(t\right)\frac{\partial f\left(x\right)}{\partial x}+\alpha \delta \left(t\right)\frac{\partial f\left(x\right)}{\partial x}$$

The resulting intensity after magnifying by factor *α* is:

(9) $$\hat{I}\left(x,t\right)=f\left(x+\left(1+\alpha \right)\delta \left(t\right)\right)$$

The LVM method is characterized by the limitation arising from the fact that the excessive strengthening of α may result in the over bumping effect—line IP4 that is visible in the final image as artefacts. Another significant disadvantage of the linear approach is the amplification of noise along with the amplification of the correct signal. For the image decomposition, LVM applies the Laplacian pyramid technique to consider the scale effect. Fundamentals of it can be found in [33,34]. To reduce the spatial noise, initial filtering with the Gauss filter is also optionally applied, which consequently yields the Laplacian of Gaussian (LOG) combination.

**Figure 3:**
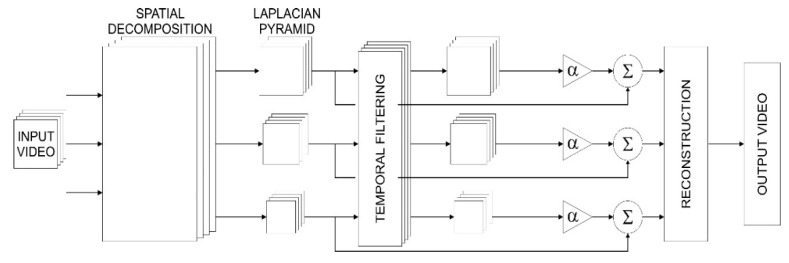
Diagram of LVM processing.

The process of LVM conversion, presented in Figure 3, includes the spatial decomposition of each frame of the input image by the Laplacian pyramid, or the Laplacian of Gaussian (LOG), temporal filtering within each layer of the decomposed images, amplification, the addition of an amplified signal to the original signal, and the reconstruction of sequence images to the output video.

### 3.3. Eulerian Phase Based

A different approach that significantly reduces the aforementioned limitation of the LVM is directed towards the intensity signal in the spatial domain from the Fourier’s perspective. The distribution of brightness intensity, described at the points with coordinates x, y, is replaced by a description of the spatial frequency as a set of sinusoidal signals of which the image is comprised. The shift of the brightness distribution corresponds, in accordance with the Fourier shift theorem, to the shift of the phase of the signals describing it. The concept of observation of the motion in an image based on the change in the signal of intensity phase in subsequent frames of the video sequence is presented in [17].

In the visual approach to diagnostics, it is usually of importance to distinguish specific components of objects which exhibit a relative movement in relation to the others, such as individual parts of a machine, or a fragment of an industrial installation surrounded by a stable background. Assuming that the expected results of the observation take into account the distinguishing between the motion of various image components, phase-based techniques refer to the local intensity signal in the form of its local phase. The main directions regarding the approach to the determination and modification of the local phase are based on the complex steerable pyramid and Riesz transform and monogenic signal [35], which is the extension of the term analytical signal to 2D. The term “steerable pyramid” was introduced in [36], based on the term of steerable filters [37], i.e., a class of filters of arbitrary orientation synthesized as a linear combination of the set of basic filters. The term “steerable” was used to indicate the shiftability in orientation [38]. Filter bank breaks each frame of the video into complex-valued sub-bands corresponding to different scales and orientations. The details can be found in [37,39]. The base function is the quadrature pair of complex sinusoids. In view of the locality in space, it is windowed by the Gaussian envelope.

In 1D space, it could be expressed by basis function modelled [11] as (10):

(10) $$e^{\frac{-x^{2}}{\left(2\sigma^{2}\right)}}e^{-i\omega x}$$

where the first part in expression (10) is gaussian function and the second is complex sinusoid.

Translation of the signal with δ take the expression (11):

(11) $$e^{\frac{-{\left(x-\delta \right)}^{2}}{\left(2\sigma^{2}\right)}}e^{-i\omega \left(x-\delta \right)}$$

The relationship between the standard deviation σ of the gaussian envelope and the frequency of the complex sinusoid ω is constant in the steerable pyramid, presented in [40,41]. The effect of motion magnifying is obtained by shifting the phase at its unchanged amplitude, which means the multiplication of the phase difference by the α coefficient. An illustration of this in 1D space can be expressed with Equation (12):

(12) $$\underset{\omega }{\sum }A_{\omega }e^{i\phi_{\omega }+\left(1+\alpha \right)\omega \delta \left(t\right)}e^{-i\omega x}=f\left(x-\left(1+\alpha \right)\delta \left(t\right)\right)$$

**Figure 4:**
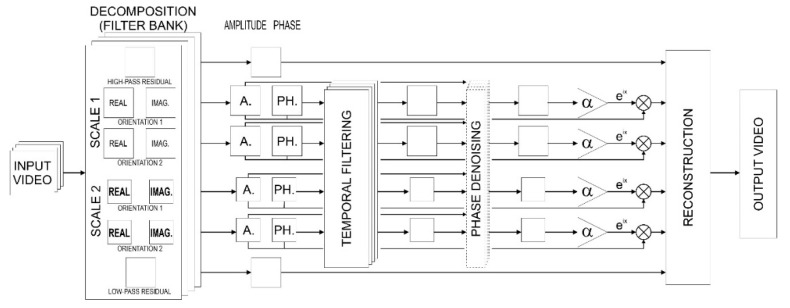
Scheme of phase-based processing.

The process of phase-based video motion magnification, presented in Figure 4, includes the decomposition and filtering of subsequent images to the form of pyramid, and the separation of the phase and the amplitude, temporal filtering the phase at the location, orientation and sale, spatial smoothing of phase to increase SNR, and the amplifying and reconstruct video sequence.

In contrast to the LVM, the maintenance of an amplitude that remains unchanged in processing enables the application of considerably greater amplifications without visible accompanying adverse phenomena such as over bumping, as well as avoiding the noise amplification since, from this perspective, they are translated and not amplified. The improvement in the actual range of noise amplification and reduction in relation to the LVM was, however, compensated by a significant increase in the computational complexity. This limits the possibilities for using it in real time. This predisposes the phase-based MM to an assessment of the technical condition at the post-processing stage. In regard to complex technical objects, where their components are characterized by the varying nature of vibrations, the application of the temporal narrowband filtering allows them to be clearly distinguished from any part of the recorded image.

The advantages associated with the use of the local phase properties in the modification of the visual recording are a source of subsequent studies in the MM area. Examples of local phase modelling and their application in image processing were presented in [41]. The most popular alternative to the phase-based methods (PBM) using the Steerable pyramid is currently motion processing with the Riesz Transform. The mathematical foundations of this group of methods derive from the term, Hilbert transform, within the 1D space, described as formula 13. The formation of an analytical signal in the form of a composite sum of the signal and its Hilbert transform allows the information on the momentary phase I of the signal amplitude to be obtained. Of the attempts to generalize the analytical signal to 2-D presented in [42], it was the monogenic signal introduced in [35] that took on a significant importance in image processing. From this perspective, an equivalent of a Hilbert transformation is the Riesz transformation. In 1D space, the transformation characterized by transfer function is:

(13) $$H\left(\omega \right)=\frac{-j\omega }{\left|\omega \right|}$$

In 2D space the transformation is characterized by formula (14) [42,43].

(14) $$H_{x}\left(\omega \right)=\frac{-j\omega_{x}}{\left\|\omega \right\|}\;\mathrm{and}\;H_{y}\left(\omega \right)=\frac{-j\omega_{y}}{\left\|\omega \right\|}$$

where *ω* is the spatial frequency.

Significant guidelines concerning the monogenic signal are also included in [44]. The use of Riesz transform in 2D is similar to the case of the analytical signal for 1D results in obtaining information on the signal phase in the spatial vision [45]. Representations of an image in the form of a Riesz pyramid in the MM process were proposed in [46]. The presented approach decomposed the input image to non-oriented sub-bands corresponding to different scales and subjected each sub-band to Riesz transformation. In order to minimize the processing duration, Riesz transform was approximated using the three-tap infinite difference filter. An alternative approach using the Quaternion Representation of the Riesz Pyramid in MM is presented in [47].

The presented methods, such as the linear Eulerian visual magnification (EVM), PBM, Riesz, referring to local changes in the image, are based on the assumption of the presence of small single motions. Under actual conditions, on complex technical objects, the small vibrations that are interesting from a diagnostics perspective occur in the presence of large shifts which, consequently, result in the occurrence of numerous artifacts and overshadows. In [48], the dynamic video motion magnification (DVMAG) technique, based on the layer magnification, was proposed. In the first stage of this method, the discount large motion is implemented by warping, using either Kanade–Lucas–Tomasi tracking (KLT) or the optical flow. In regard to the magnifying motion, the second stage uses the phase-based Eulerian magnification in the layers of foreground and opacity in every video frame, obtained by the alpha-matte. The holes revealed following the reconstruction are filled using the texture synthesis. The disadvantage of the presented method is the necessity of manual selection of the region of interest (ROI) [49]. The modified alpha-matte technique presented in [50] decreases the user interactions occurring in the DVMAG. The video acceleration magnification technique, presented in [51], eliminates the need for the explicit estimation of motion by obtaining the spatial acceleration magnification by the application of temporal second-order derivative filtering, with deviation of changes in video (only with linear large motion).

The magnification of small motions in the presence of nonlinear large motions [52] with amplitude-based filtering was presented in [53]. The cited study provides for the increase in small movements, while maintaining the large ones unchanged. In the modified linear EVM after the decomposition of each video sequence frame into a spatial pyramid in every spatial layer, the time series of intensity differences between the subsequent points on the time axis at the corresponding space positions were determined. The obtained discreet signals were transferred to the frequency domain and considered to be as a sum of two components with spectral amplitude above and below the adopted threshold. In order to eliminate large motions, an amplitude-based filtering with a two-valued weighting function was performed. Spectral amplitude exceeding the adopted threshold determines the value of the weighting function as 0, while in the remaining cases it amounts to 1. The cited study adopted a threshold equal to the smaller of the two: mean or median of the amplitude. Following the filtration, small signals in the spatial layers are magnified and, ultimately, the pyramids are collapsed. In the phase-based Eulerian approach, the amplitude-based filtering is used for the variation phase instead of the intensity difference variation. An alternative approach for the magnification of small motions in the presence of large motions using convolutional neural network is presented in [54].

The technique presented in [55] expands the area of applications of video motion magnification to include observations of small motions in the presence of quick, large motions. The leading subject of the cited study is the elimination of the effect of quick, large motions expressed with significant changes in the acceleration. This phenomenon, referred to as “jerk” and described as the third derivative of movement in the function of time, can be a measure of the assessment of time series data in terms of smoothness. The solution presented in the article involves the design and application of a jerk-aware filter (JAF), which cuts off quick large motions. The magnification of small motions is carried out using the phase-based acceleration technique and steerable pyramid to decompose input video frames.

The potential problem affecting the quality of the final effect of the MM is not limited to the presence of motions described above as large in an image. Natural phenomena that accompany the recording of an image coming from a light source or the operation of a sensor may cause an effect of the appearance of changes, which are similar to changes meaningful in regard to their real information concerning the condition or behavior of the object under observation. The mixing of meaningful and non-meaningful subtle changes results in the emergence of significant noise in the magnified image. Apart from the recognized techniques for minimizing this effect, related to the manual intervention into to the processing process, a method using the so-called factional anisotropy (FA) was proposed in [56]. The FA, used in neurosciences, was used in the cited study to design a filter eliminating non-meaningful changes. The idea of using FA is based on the observation of temporal distribution of changes, which, for the meaningful ones, demonstrate a clearly anisotropic nature of diffusion in contrast to the non-meaningful changes. The MM process presented in the study, which adopted the phase-based FA filter, was performed with the jerk-aware phase-based method.

## 4. Examples

The area of MM applications is becoming increasingly wide. Selected areas of application such as medical analysis of physiological signals, modal analysis of structures, micro expression recognition, visual sound recovery or visual vibrometry were indicated in, for example, [57]. Other examples of MM applications, drawn from the literature, include the investigation of changes in the vibration behavior of ductile iron pipes occurring due to corrosion [58]. In [59], an analysis of phase-based MM operational deflection shapes of a wind-turbine blade was carried out for both a healthy and damaged structure. In [60], the applications of EVM for detection in civil engineering structures bonded with fiber-reinforced polymers are presented. Modal vibration examination and the damage detection of antique structures using MM are presented in [61]. In [62], light poles located on an elevated highway bridge were subjected to modal analysis, using MM to amplify the micro-vibration of its structure. An example of MM application for multipoint modal identification in civil structures and construction (pedestrian bridge) is presented in [63]. The results of laboratory tests of modal identification of a cantilever are presented in [64]. In [65], the results of an experiment are also presented for a pipe cross-section. In the case of the experiment presented in [66], broad-band filtering motion magnification (BPMM) was used to observe aerodynamically induced nozzle vibration. In [67], the scope of the modal analysis was extended to include tests for damage identification using genetic algorithms (GA). The effects of testing the shell of cylindrical structures with no weld lands using the flow-formed manufacturing process are covered in [68]. The intuitive nature of the observation of the vibration of a tower under horizontal load amplified with MM was analyzed in [19]. A more complex procedure for a dynamic structural system identification is proposed in [69]. The presented approach, including pre-modification of an input video stream and post-modification of the magnified video, was verified by examining the vibrations of a minaret spire. Pre-modification included such operations as rotation, crop, zoom and increasing the video contrast value to the maximum level. Post-modification was carried out to remove the magnification-generated waves. In the prototypical implementation of a vibration sensor [70], MM is used for sensing the motion of spider silk. An image of the motion of spider silk was magnified with Eulerian phase-based video motion processing.

In the next part, selected examples of diagnostic tests, supported by the video stream processing technology using motion magnification, which were obtained from resources of the authors professional team, involved diagnosing the condition of technical objects. The reasons for undertaking the tests including the examples were inspired by the abnormal operation of devices reported by maintenance, as well as maintenance activities in accordance with the routine service schedule. In each of the presented examples, the recorded image was subjected to MM amplification using commercial equipment and software. Observation of the explicit effect of enhanced displacements of the positions of interacting components can be seen when playing a video sequence. The actual effect of the movement of moving components focuses the observers’ attention on the significant components that may be difficult to notice in still images. In the presented illustrations, they were exposed regions of pictures. They point to the components of devices, the behavior of which enables the identification of the emergent problem. Complete video material is available at: https://bit.ly/39qgZGT (accessed on: 30 September 2021).

**Figure 5:**
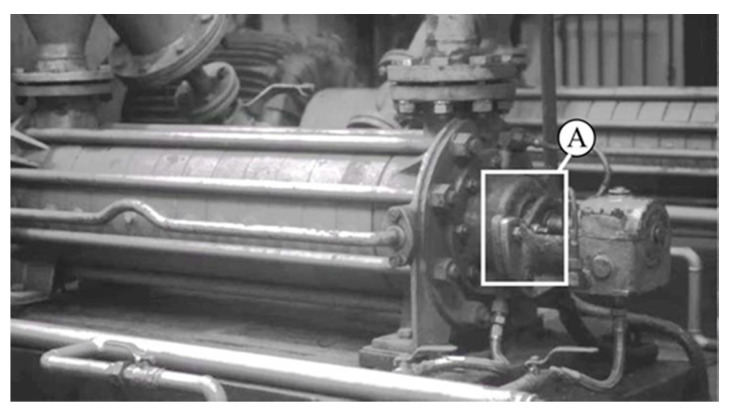
Supply pump.

**Figure 6:**
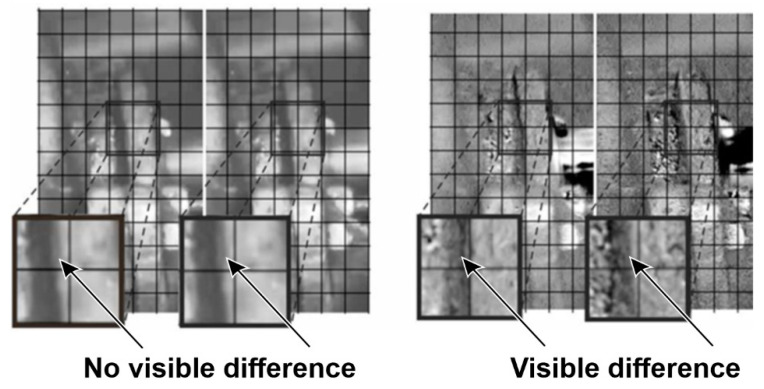
Selected video frames presenting the displacement of pump seals (magnification of the areas indicated in Figure 5 as A). Unprocessed images are shown on the left, and images processed with the use of the motion amplification method are shown on the right.

In the case of the supply pump presented in Figure 5, a routine condition inspection of the supply installation revealed excessive vibrations of the casing and the installation interacting with it. The tests revealed defects in the assembly of the seal. Video frames presenting the axial movement of the pump mechanical seal clamps and bearing housing are shown in Figure 6. The effect of processing was illustrated in the pairs of windows. The left side shows two adjacent, unprocessed views of the seal casing, corresponding to two different locations of the pump shaft. In this case, the images appear to be identical. The views on the right are their processed versions. In this case, the different locations of the dark edge of the casing in relation to the marked grid (visible in the neighboring zoomed fields) reveal its changing location.

**Figure 7:**
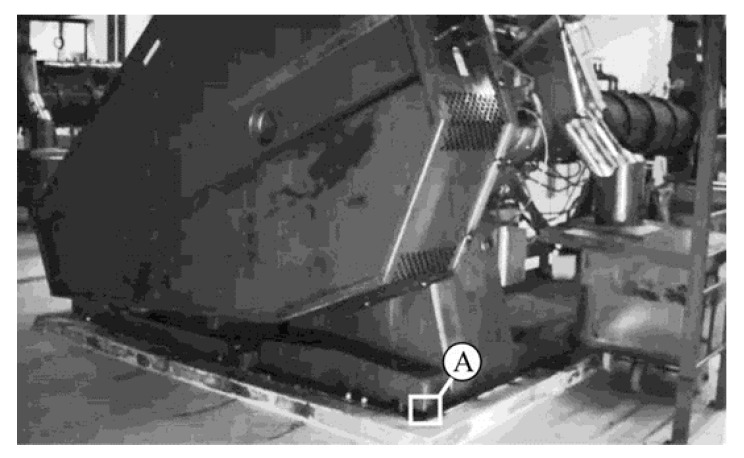
Dry feed extruder.

**Figure 8:**
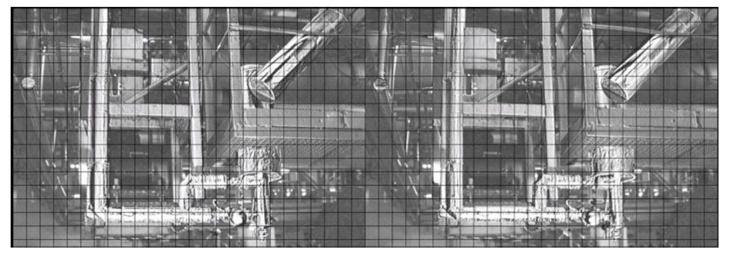
Accompanying installations of dry feed extruder.

**Figure 9:**
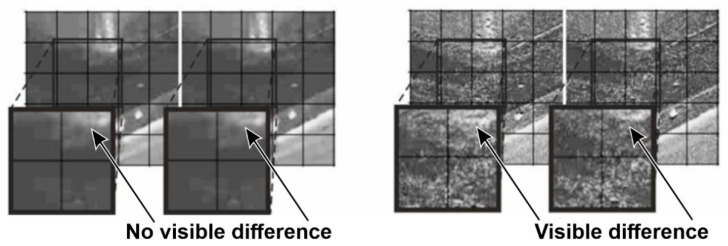
Selected video frames presenting the elements mounting the extruder to the foundations (magnification of the areas indicated in Figure 7 as A). Unprocessed images are shown on the left, and images processed with the use of the motion amplification method are shown on the right.

In regard to the dry feed extruder shown in Figure 7, the original reason for undertaking the tests was the abnormally high vibrations of the extruder casing and the structural equipment of the object shown in part in Figure 8. The observation of MM-enlarged video recordings demonstrated the assembly failing, resulting in movements of the component attaching the extruder base to the ground, presented in Figure 9. The applied visualization diagram is analogous to that in Figure 6.

**Figure 10:**
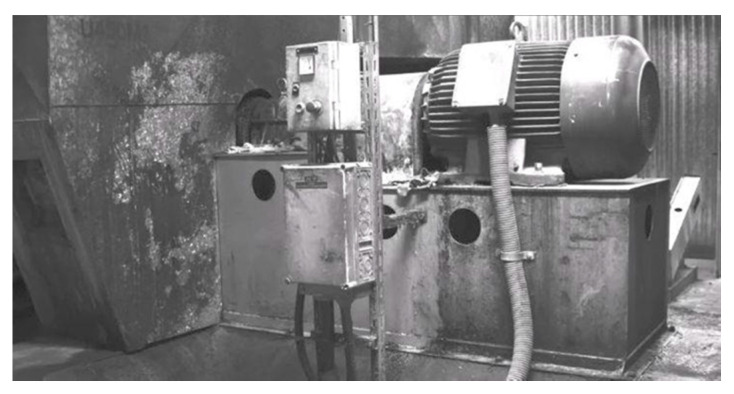
Fan drive.

The third example is an effect of the actions undertaken as a result of intensified vibrations of the floor and the machine hall ventilation fan casing, presented in Figure 10.

**Figure 11:**
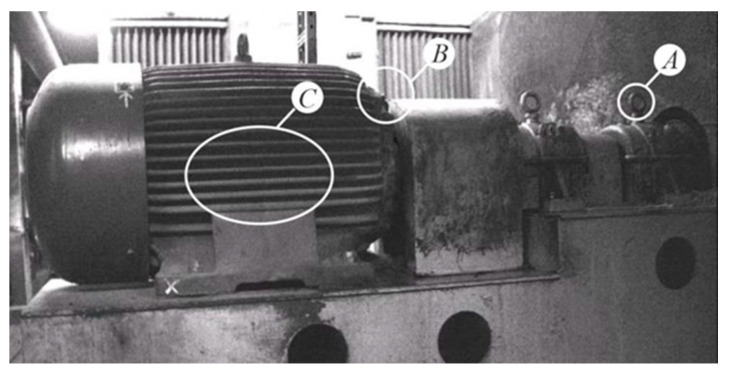
Motor/fan drive connection.

**Figure 12:**
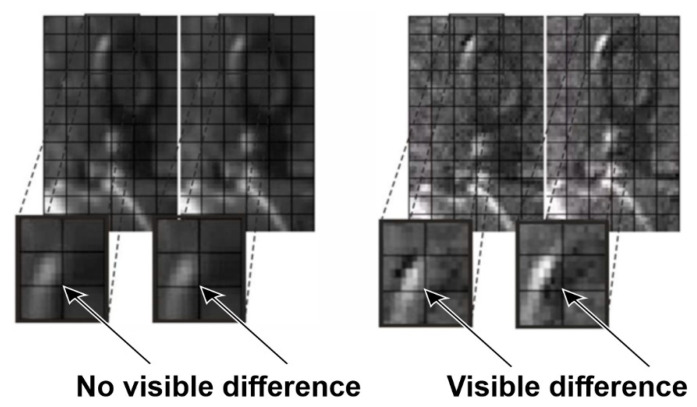
Selected video frames presenting the bearing housing displacement in the fan’s drive shaft (magnification of the areas indicated in Figure 11 as A). Unprocessed images are shown on the left, and images processed with the use of the motion amplification method are shown on the right.

**Figure 13:**
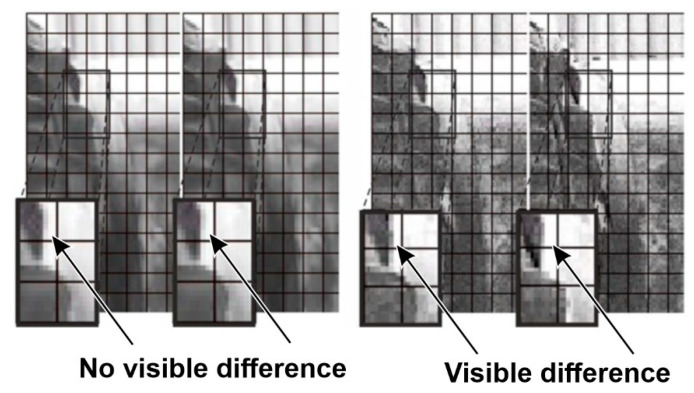
Selected video frames presenting the motor casing displacement on the side of the clutch (magnification of the areas indicated in Figure 11 as B). Unprocessed images are shown on the left, and images processed with the use of the motion amplification method are shown on the right.

**Figure 14:**
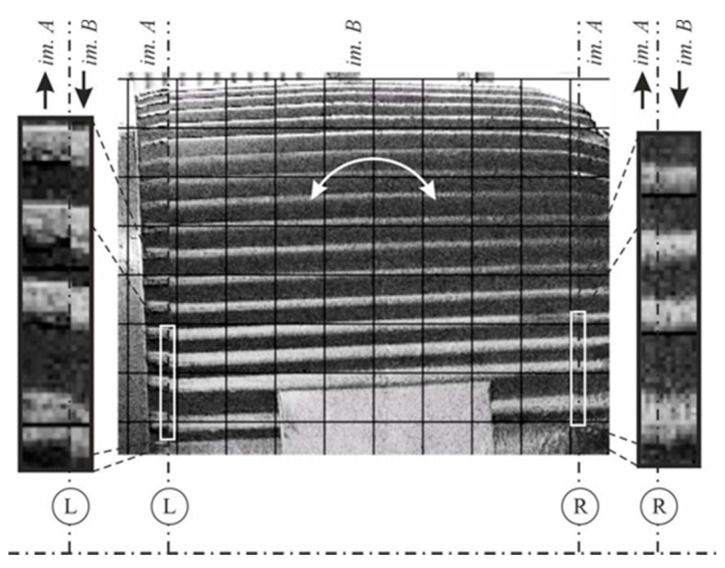
Selected video frames present rotation of motor body. It is visualized as magnification of compound of selected parts of images (Im A and Im B) in different frames (areas indicated in Figure 11 as C).

The fluctuations of locations of individual components of the power transmission chain from the engine shaft to the ventilator shaft, exposed by the use of the MM, indicated the misalignment errors in the assembly of the coupling. Changes in the position of individual parts are shown in Figure 11, Figure 12, Figure 13 and Figure 14. The applied visualization diagram is analogous to that in Figure 6.

## 5. Discussion

The most common among the presented MM methods are Eulerian methods. Due to the very diverse area of applications of the successive modifications of these techniques, their direct comparison is much more difficult. The difficulties related to the different nature of the object’s movement and its environmental context also concern the monitoring of a technical objects’ condition. In this situation, it is much more difficult to adopt an unambiguous criterion for the assessment of the processing effect achieved and the methodology applied. The original aim of the MM (and more broadly, video magnification of VM) methods is to overcome/break the boundary of human perception of changes (displacement) of the objects observed. The results of the developed algorithms also include other functionalities of these methods, thus enabling quantitative estimation of the kinematic parameters of the objects observed. In such cases, the evaluation of the method can be carried out in terms of compliance of the data obtained with the data derived from alternative measurement systems.

In [11], the results of processing the image of cantilever beam vibrations by the phase-based method, obtained under laboratory conditions, were compared with the laser vibrometer results. The effect of comparison was expressed using the value of correlation between displacement values (at a level above 0.99). In [40], similarly to [11], vibrations of the beam induced with an impact hammer were recorded. A comparison of the MM result was referred, in this case, to the signal from the accelerometer, and the result was expressed as L1 error estimation. The verification of results based on the data from the accelerometer and the camera was also applied in [71]. The comparison was made under laboratory conditions using an accelerometer and a camera located at a distance of 1.2 m from a vibrating cantilever beam (impacted with a hammer). The difference determined in the displacements of selected sources was at a level of 0.05–0.14%. The experiment was also conducted under real conditions on a weighted bridge, this time showing the differences between the results obtained in the form of frequency differences for individual modes at a level of 0.7–3.9% (within the frequency level of 2.7–7.0 Hz). A comparison of phase-based MM data with the data from the accelerometer and laser vibrometer is presented in [65] in an application for modal analysis. A comparison of the achieved displacement signal when using a rapid camera (5000 frames/second) and a laser vibrometer was expressed by the correlation value (99.6%) and a table of values used in the modal analysis of the modal assurance criterion (MAC) parameter.

Besides the accelerometer, an additional source of a reference signal for the assessment of structural health monitoring of a bridge structure in [72] was a strain gauge sensor. The study also suggests the use of a Kalman Filter, based on the fusion of data from the accelerometer and MM, to generate a signal that was compared with results of numerical model analyses. However, the main aim of motion magnification (which is mentioned in many publications along with color magnification; an example of simultaneous color and motion magnification can be found in [73]) is the visualization of movements in a recorded image that are not visible to the naked eye. Where the object’s condition is assessed, it provides initial (ad hoc) information on changes in the object’s geometry, which are significant in terms of the device operation. In this case, the assessment of the method/technique used is determined by different criteria. They refer both to the perceptual process itself and to the desirable effect of observing phenomena of a diverse nature. The presentations of results using image quality measures derived from classical image and video processing methods are included inter alia in the studies [74].

The measures most often used due to the simplicity of implementation include the mean squared error (MSE) and peak signal-to noise-ratio (PSNR). Another parameter frequently used in the image quality evaluation is the structural similarity (SSIM) index [75], which combines the reference to luminance, contrast and structure. Paper [46] presents a quantitative comparison of the Eulerian MM method based on the sequence of a synthetic oscillating Gaussian, in which the ground-thru motion sequence is known. Within the range of the amplification factor of up to 200, the RMSE value was similar to Riesz and CSP, with a considerably deviating RMSE value in the case of a linear approach. A similar nature of divergence was obtained for RMSE as a function of (σ) noise value. A comparison based on PSNR and SSIM was used in [76] to illustrate the effect of noise reduction using the Combined Wavelet Domain Spatio-Temporal Video De-Noising by the block-based motion detection method. In this case, the ground-thru, as an image subjected to MM, was compared with an image corrupted with Gaussian noise also subjected to MM. An example of an increase in PSNR at (σ) noise deviations 20 was from x1.9 (baby) to x1.4 (wrist). Paper [77] shows the advantage of, inter alia, the phase-based method over the linear method by presenting numerous image quality measures, including the MSE, PSNR and SSIM values, averaged for different images (a baby, guitar, etc.), without providing the source of ground-thru.

The presented methods for determining quality measures referring to the ground-thru are classified as full-reference. In the case of motion magnification, this presents a significant difficulty in view of the fact that the desirable image deformation, specific for this method, prevents a direct comparison of its parts on the corresponding positions of the input and output (processed) image. Quantitative measures of the effect of noise reduction with wavelet processing, including MM and CM, and presented in [78], were determined by comparing an original and a magnified image in relation to color variations.

An alternative way to quantitatively describe the image quality is to apply methods from the no-reference group. In such cases, BLINDS techniques [79], which refer to the human perceptual evaluation of an image, are applied. The Index differential mean opinion score (DMOS) based on this approach is presented in [50] to illustrate an increase in image quality, thanks to the application of the interest region-based motion magnification method, in relation to classic EVM. The inclusion of the interaction with a human observer to the assessment was applied in [80] to optimize the video magnification process parameters. An example of the improvement of image quality due to a correction of the amplification factor in relation to those suggested in other studies, presented in the study, takes place after approx. 15 user evaluations. In most published studies, besides the quantitative quality measures, a highly effective method of presenting effects/results is to attach processed images to the text, which leaves room for interpretation by the reader/viewer. A classic source of input images are the video materials originally used by authors from MIT, e.g., a video of a sleeping baby, vibrating guitar strings, etc.

An important criterion for comparing MM techniques in terms of their ability to be implemented online is the computational complexity of the algorithms used. This determines the duration of the processing, although it is often in conflict with other requirements in relation to the desired processing result. The value of the time-consuming parameter that characterizes Eulerian methods in their basic version can be expressed using the relationships presented in the Table 2.

Table 2, based on numerous studies, is a certain useful simplification, resulting from the implementation details of the algorithms used. For example, [81] suggests reducing the processing duration when applying the Riesz method by 4–5 times, in relation to complex steerable without compromising the quality. An original source [46] indicated these values for the extreme variants of the tested algorithms, concerning eight orientation complex steerable pyramids and approximated Riesz transforms based on three-tap finite difference filters. The summary presented in the study suggests that the application of Riesz transforms approximation alone in place of the Riesz transforms in the frequency domain reduces the computation time by approx. 25–30%. Similarly, the limitation from 8 to 2 directions of complex steerable considerably changes the result of the comparison. This issue is also indicated in [18]. Paper [82] also draws attention to the achieved processing effect, pointing out to the change, in the case of an approximation of Riesz, in the number of artifacts in relation to the use of Riesz based on FFT.

An increase in the duration of processing accompanies the modifications of MM algorithms introduced at the post-processing stage. The enhanced Eulerian video magnification (E2VM) [83] motion analysis based on 2D Motion mapping applied in the technique results in an increase in the processing duration by 14.5% compared to the classic Eulerian-linear algorithm. However, the result obtained is also a result of simplifications that involve subsampling at the motion mapping stage. A negligible increase of the processing duration at a 3% level accompanies the wavelet denoising method, applied in post-processing between the stages of phase magnification and image reconstruction. Using the sparse representation proposed in [84] reduces the computational time to 1/5 of the traditional method by reducing the number of filters from 1200 to 345. Attempts to provide a tabular summary of the selected characteristics of the EVM methods were made, for example, in [77].

The cited differences in the processing rate, resulting from the complexity of algorithms, are related to the software layer of developing tools for visualizing motion in an image. The hardware layer used has an equally significant effect on the processing duration, thanks to the increasingly efficient computational units and dedicated hardware architecture solutions. An example of a significant increase in the efficiency of pixel parallel EVM in relation to classic solutions is presented in [85]. The diverse nature of both the phenomena observed using MM and the immediate vicinity in which they occur is a factor determining the optimization of their algorithms that is as significant as striving for noise level reduction. Depending on the specific conditions, these issues are addressed by the studies cited in Section 3.3. Paper [86] extends the applicability of phase-based video motion magnification (PVMM) limited to stationary vibration measurement to include measurements of non-stationary vibration using Time-Varying Motion Filtering. Regarding non-stationary vibrations, this approach generates fewer artifacts and lower noise. Selected MM methods according to the fit criterion for the application are presented in Table 3.

## 6. Conclusions

The growing number of practical applications of the MM methods, implemented by maintenance services under real industrial conditions, confirms the effectiveness of the presented methods while supplementing traditional diagnostic procedures. In addition to the undoubted effects of maintaining the proper condition of the equipment based on a visual assessment of vibrations applying the MM methods cited in the article, the MM also enables a quantitative assessment of motion parameters. The presented examples of the implementation focus on the perceptive measures aimed at the rapid location of the problem occurring in the object, rather than on the complex parametric description. In this context, the negative phenomena accompanying the MM process, such as the formation of artefacts, can, in certain situations, make it easier for the observer to capture significant details that distinguish them from the wider background. Having accepted the basic fact that the final outcome of the MM technique is the obvious deformation of the reality being recorded, it appears beneficial to further develop these methods, not only to improve the image reality resemblance but also to contrast disclosed points of interest. The aspect of relaxing requirements in regard to the computational complexity emphasized in the MM research, leans towards the possibility of real-time processing. The reduction in time at the post-processing stage is of particular importance in the event of on-going observation of the object. In many cases, the clear manner of distinguishing the deviation from the desirable condition of the object is of equal significance, which can determine further directions of research into the application of MM under the conditions of an actual industrial environment.

## Figures and Tables

**Table 1:** Comparison of base constrains of accelerometer and visual motion sensor.

Accelerometer	Video Camera
Contact	Contactless
Sparse discrete pointwise measurements	Simultaneous multi point location (quasi continuous)
Close neighborhood impact in assembly point (sensitivity to temperature, chemicals, etc.)	Line of sight disturbance sensitivity (sensitivity to lighting, fog, smoke, etc.)
Measurement of absolute values	Measurement of relative values (relative to camera base)
Direct acquisition	In plane 3d to 2d projection (in case of single camera)

**Table 2:** Comparison of general features of various Eulerian methods.

Time consumption	linear	<	phase-based Riesz	<	phase-based complex steerable
Loss of quality	phase-based complex steerable	<	phase-based Riesz	<<	linear
Limit of magnification factor	linear	<	phase-based Riesz	≈	phase-based complex steerable

**Table 3:** Selected motion magnification methods according to the fit criterion for the application.

Lit. pos.	Method	Recommendations
[32]	Linear video magnification	Basic fast application, low magnification fac-tor, magnified noise, numerous artefacts
[40]	Eulerian phase-based complex steerable	Postprocessing (computationally consuming), very good image quality, good noise performance, high magnification factor
[47]	Eulerian phase-based Riesz	Less computationally consuming than complex steerable, good image quality, good noise performance, high magnification factor
[20]	Spatio-Temporal Context Learning and Taylor Approximation	Condition of illumination changes and fog interference
[18]	Vibration detection based on out-of-plane vision	Motion in the direction perpendicular to the focusing screen plane
[23]	Structural displacement monitoring using deep learning-based full field optical flow	Reduction of user involvement in OF processing
[48]	Temporal stabilization followed by layer-based magnification and magnify ROI with Matting	Discount large motion, necessity of manual selection of the region of interest (ROI)
[53]	Improved Linear EVM algorithm using amplitude-based filtering.	Small motions in the presence of large motions
[55]	Application of a jerk-aware filter	Small motions in the presence of quick large motions (jerk movement)
[56]	using the factional anisotropy	Eliminating non-meaningful changes (e.g., neuroscience)
